# Practical Departments

**Published:** 1904-01-16

**Authors:** 


### PRACTICAL DEPARTMENTS.

### A USEFUL FILE.

We have received a sample of a file that has decided advantages, as it serves as an index as well as a manuscript store, for a very reasonable price. The file is a neat and well-made box, provided with inner portfolios initialled for every letter of the alphabet, the whole compressed and secured by a firm but easily moved spring clip. The box is fastened by a simple spring hasp, and the papers can be easily examined without removal. To the back of the file a ring is attached, so that when it is placed on the shelf it can be easily extracted, which, when ordinary boxes are crowded together is not the case. We can really recommend these files, which are supplied by the Ideal Syndicate, 2 Pancras Lane, Queen Street, E.C., who have also a series of most useful and inexpensive cabinets for containing the files and other conveniences for the use of busy people. The Syndicate have specialities of all kinds for office use.

